# A meta-analysis of the American college of surgeons risk calculator's predictive accuracy among different surgical sub-specialties

**DOI:** 10.1016/j.sipas.2024.100238

**Published:** 2024-02-13

**Authors:** Alyssa M. Goodwin, Steven S. Kurapaty, Jacqueline E. Inglis, Srikanth N. Divi, Alpesh A. Patel, Wellington K. Hsu

**Affiliations:** Department of Orthopaedic Surgery, Northwestern University Feinberg School of Medicine, Chicago, IL 60611, USA

**Keywords:** Surgical risk calculator, Complications, Surgical sub-specialty

## Abstract

**Background:**

The American College of Surgeons National Surgical Quality Improvement Program (ACS-NSQIP) provides risk estimates of postoperative complications. While several studies have examined the accuracy of the ACS-Surgical Risk Calculator (SRC) within a single specialty, the respective conclusions are limited by sample size. We sought to conduct a meta-analysis to determine the accuracy of the ACS-SRC among various surgical specialties.

**Study design:**

Clinical studies that utilized the ACS-SRC, predicted complication rates compared to actual rates, and analyzed at least one metric reported by ACS-SRC met the inclusion criteria. Data for each specialty were pooled using the DerSimonian and Laird random-effect models and analyzed with the binary random-effect model to produce risk difference (RD) and 95 % confidence intervals (CIs) using Open Meta[A*nalyst*].

**Results:**

The initial search yielded 281 studies and, after applying inclusion and exclusion criteria, a total of 53 studies remained with a total sample of 30,134 patients spanning 10 surgical specialties. When considering any complication and death, the ACS-SRC significantly underpredicted complications for: Orthopaedic Surgery (RD –0.067, *p* = 0.008), Spine (RD -0.027, *p* < 0.001), Urology (RD -0.03, *p* < 0.001), Surgical Oncology (RD -0.045, *p* < 0.001), and Gynecology (RD -0.098, *p* = 0.01).

**Conclusion:**

The ACS-SRC proved useful in General, Acute Care, Colorectal, Otolaryngology, and Cardiothoracic Surgery, but significantly underpredicted complication rates in Spine, Orthopaedics, Urology, Surgical Oncology, and Gynecology. These data indicate the ACS-SRC is a reliable predictor in some specialties, but its use should be cautioned in the remaining specialties evaluated here.

## Highlights

• Potential post-operative complications are important in-patient care and surgical planning.
• The American college of surgeons calculator predicts post-surgical complications.
• The calculator is utilized over many subspecialties but may not accurately predict complications.
• The calculator predicts complications accurately for certain subspecialties.

**Abbreviations and Acronyms**
ACS-SRC: American college of surgeons surgical risk calculator
CPT: current procedural terminology
LOS: length of stay
VTE: venous thromboembolism
SSI: surgical site infection
UTI: urinary tract infection
OR: operating room
RD: risk difference

## Introduction

Postoperative complications place a significant strain on health systems and patients financially [1], [2], [3]. Nearly 7–15 % of patients undergoing surgery are affected with postoperative mortality rates reaching 5.7 % [1]. Unsurprisingly, hospital costs are $19,262 higher per patient with complications compared to those without (*p*<0.001) [2]. Complication rates face further scrutiny amongst both payer and providers with the introduction of incentivized reimbursement and bundled payments [4]. Additionally, hospital systems want to remain competitive in context of cost and quality with new visibility from the Transparency in Coverage Final Rules [5]. As institutional leadership seeks solutions, validated, reliable predictive tools can provide valuable guidance [6], [7], [8].

**Fig. 1:**
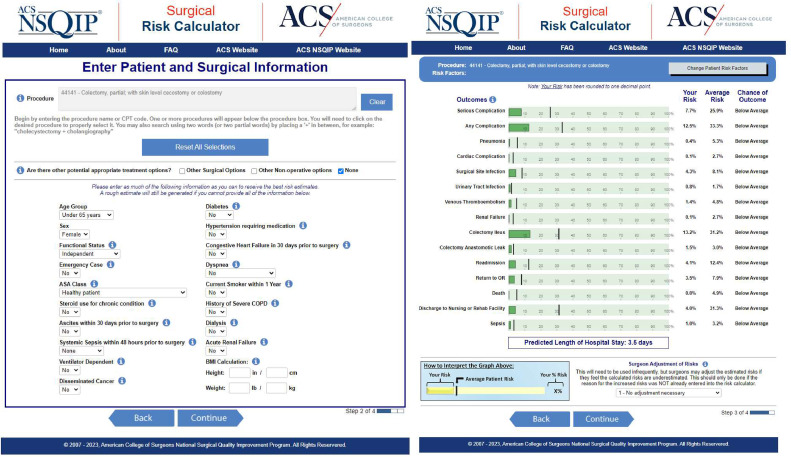
Screenshot of the American College of Surgeons Surgical Risk Calculator (ACS-SRC). A. Individual patient risk factor entry screen and B. Patient surgical outcomes report (https://riskcalculator.facs.org/RiskCalculator/).

The calculation of a risk-benefit ratio associated with surgery is integral in shared patient-physician decision-making [1]. One widely used tool for the prediction of postoperative complications is the American College of Surgeons Surgical Risk Calculator (ACS-SRC) [1], powered by the National Surgical Quality Improvement Program database (NSQIP). The ACS-SRC was first released in 2013 and has evolved to include data from over 5 million operations with 874 participating institutions [1]. The calculator requires the input of nineteen patient specific variables with a relevant current procedural terminology (CPT) code and predicts rates of 14 different post-operative complications: serious complication, any complication, pneumonia, cardiac complication, surgical site infection (SSI), urinary tract infection (UTI), venous thromboembolism (VTE), renal failure, readmission, return to operating room (OR), death, discharge to nursing or rehab facility (adverse discharge), sepsis, and length of stay (LOS) [9]. Additionally, the calculator reports patient risk compared to an average patient risk and compiles a chance of outcome column for each complication (Fig. 1). It remains unclear, however, if the calculator is accurate across different surgical subspecialities.

Our study seeks to quantify and compare the predictive accuracy of the ACS-SRC and its performance among surgical specialties through a standardized meta-analysis protocol. While many studies have reported results from specific procedures in one specialty, no publications to date have quantified the effect size or correct for single-institutional biases [9,10]. This study is the first to address the accuracy of the ACS-SRC across multiple surgical specialties.

## Methods

**Fig. 2:**
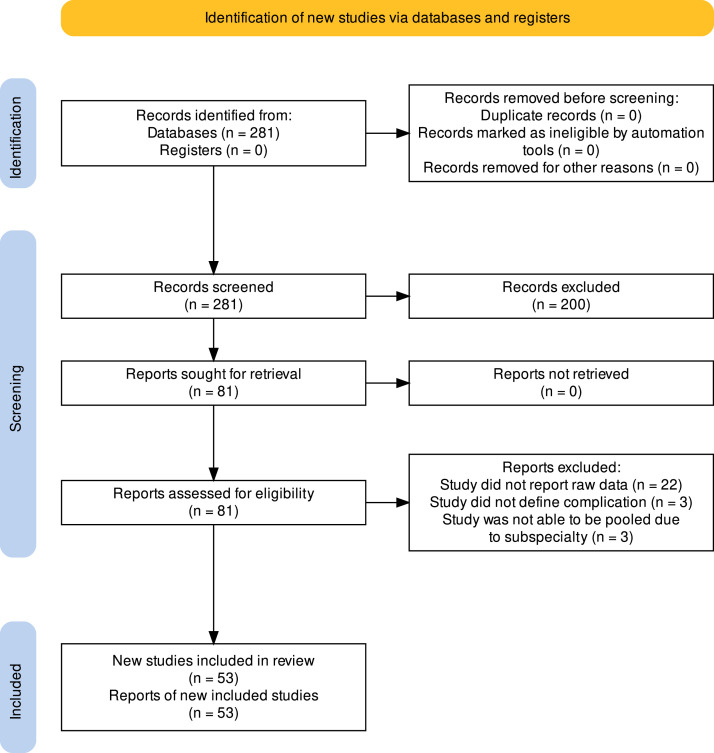
Prisma Flowchart. Chart that illustrates the study selection process, the number of studies screened and assessed, and the final number of studies included in the meta-analysis.

A literature search was performed of the PUBMED database on March 15, 2023. The terms searched included “surgical risk calculator”, “American College of Surgeons surgical risk calculator”, and “NSQIP”. Inclusion criteria for clinical studies required the following: comparison of ACS-SRC predicted complication rates compared to actual complication rates as reported by ACS-SRC (Fig. 1), clear definition of type of complication, and data presented as raw data or percent with sample size provided. Studies were excluded if they only reported Brier scores, C-statistics, or area under the curve (AUC) (Fig. 2). The standardized definition of “any complication” includes SSI, wound disruption, pneumonia, unplanned intubation, PE, ventilator > 48 h, renal insufficiency, acute renal failure, UTI, stroke, cardiac arrest, myocardial infarction, DVT, return to OR, and systemic sepsis. Of note, “any complication” does not include death, readmission, LOS, or discharge to nursing or rehab facility (Fig. 1). In this study, any complication and death were aggregated to comprise a comprehensive complication rate for each specialty. A specialty was only included in the analysis if at least two articles reported complication rates. Studies were not filtered by sample size or population characteristics. This metanalysis was registered in Prospero with the ID of CRD42023473595.

### Statistical analysis

Data for each specialty were pooled by the log-transformed event rates using the DerSimonian and Laird random-effect models. All analyzes were performed using a binary random-effect model to produce risk difference (RD) and 95 % confidence intervals (CIs). Heterogeneity was assessed using the *I*^2^ statistic. This statistic represents the proportion of variability that is not attributable to chance. *I*^2^ values >50 % indicate substantial heterogeneity. Statistical analyzes were conducted using Open Meta[Analyst] Windows 10 [11]. All values were two tailed, and a *P* < 0.05 was set as the threshold for statistical significance.

## Results

The initial PubMed search yielded 281 studies which was reduced to 81 studies after full-text review. After applying inclusion and exclusion criteria, a total of 53 studies remained. A total sample of 30,134 patients spanned 10 surgical specialties: General Surgery, Spine Surgery, ENT, Urology, Cardiothoracic (CT) Surgery, Colorectal Surgery, Acute Care Surgery, Oncological Surgery, Gynecological Surgery, and Orthopaedic Surgery. “Spine Surgery” included both articles published in Neurosurgery and Orthopaedic journals (Table 1).

**Table 1:** The Number of Studies Included for Each Surgical Field.

Fields	Number of Studies
Cardiothoracic	3
Colorectal	3
Emergency/Acute Care	2
ENT	7
General Surgery	9
Spine Surgery	7
Oncology	6
Orthopaedic Surgery	4
Urology	6
Gynecology	5
Grand Total	53

**Fig. 3:**
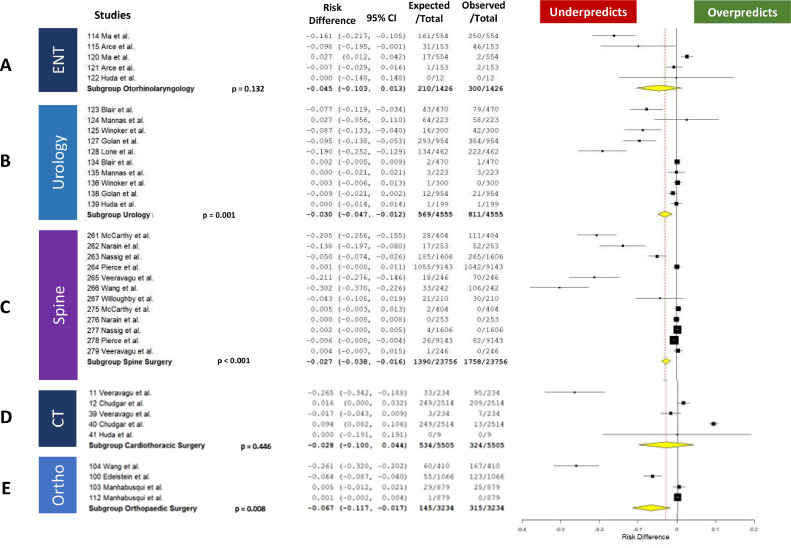
Forest Plots Showing the Risk Difference between the ACS-SRC Predicted and Patient Observed Values by Surgical Specialty. A. ENT, B. Urology, C. Spine. D. Cardiothoracic (CT), E. Orthopedic Surgery (Ortho).

**Fig. 4:**
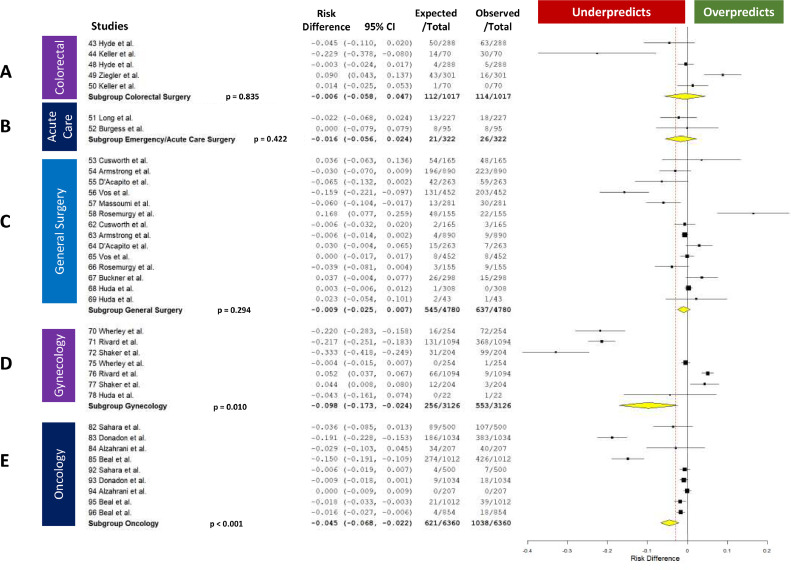
Forest Plots Showing the Risk Difference between the ACS-SRC Predicted and Patient Observed Values by Surgical Specialty. A. Colorectal, B. Acute Care, C. General Surgery, D. Gynecology, E. Oncology.

When considering any complication and death, the ACS-SRC significantly underpredicted complications for: Orthopaedic Surgery (RD –0.067, 95 % CI –0.117 to −0.017, *p* = 0.008), Spine (RD −0.027, 95 % CI –0.038 to −0.016, *p* < 0.001), Urology (RD −0.03, 95 % CI −0.047 to −0.012, *p* < 0.001), Surgical Oncology (RD −0.045, 95 % CI –0.068 to −0.022, *p* < 0.001), and Gynecology (RD −0.098, 95 % CI –0.173 to −0.024, *p* = 0.01) (Figs. 3 and 4).

There was no significant difference between the actual and ACS-SRC predicted complication rate for Otorhinolaryngology (ENT) (RD −0.045, 95 % CI −0.103 to −0.013, *p* = 0.132) General Surgery (RD –0.009, 95 % CI –0.025 to 0.007, *p* = 0.294), Cardiothoracic Surgery (RD –0.028, 95 % CI –0.100 to 0.044, *p* = 0.446), Acute Care Surgery (RD –0.016, 95 % CI –0.056 to 0.024, *p* = 0.422), and Colorectal Surgery (RD –0.006, 95 % CI –0.058 to 0.047, *p* = 0.835) (Figs. 3 and 4).

## Discussion

The ACS-SRC can provide valuable insight into understanding postoperative complication rates after surgery. The current literature has few studies that assess the ACS-SRC at a specialty level [12], [13], [14] with the majority of studies focusing on procedure-specific evaluations [15], [16], [17], [18], [19]. Our work demonstrates a specialty-level view of this risk calculator to better understand its ideal use and limitations. Prior work examining the validity of the ACS-SRC only assesses predictive accuracy in a binary fashion [12], [13], [14], [15], [16], [17], [18], [19], providing a rudimentary analysis of the predictive ability of the calculator. This study reports a risk difference, allowing for both an assessment of statistical significance and directionality (under- or over-prediction of calculator).

The ACS-SRC can be an effective tool in pre-operative planning in certain surgical fields. When considering its overall ability to predict complications by specialty, the ACS-SRC proved useful in General Surgery, Acute Care Surgery, Colorectal Surgery, ENT, and CT Surgery. Conversely, the calculator significantly underpredicted complication rates in Spine Surgery by 2.7 %, Orthopaedic Surgery by 6.7 %, Urology by 3.0 %, Surgical Oncology by 4.5 %, and Gynecology by 9.8 %. In comparison, complication rates in General Surgery were underpredicted by only 0.9 %, showing accurate prediction by the calculator. These data indicate the ACS-SRC is a reliable predictor of complications in CT, ENT, General, Acute Care, and Colorectal Surgery, but its use should be cautioned in the remaining specialties evaluated here.

The accuracy of the ACS-SRC in General Surgery demonstrated by this study aligns with previous literature. Cologne et al. found that the ACS-SRC accurately predicted LOS, serious complications, and any complications rates across 116 laparoscopic colectomy patients [18]. In an octogenarian cohort consisting of both elective and emergency cholecystectomies, D'Acapito et al. reported high discriminative ability of the ACS-SRC, particularly regarding death and serious complications [20]. Additionally, Long et al. evaluated the accuracy of the calculator across emergent cases and found accurately predicted rates for all complications except for LOS [21]. Our work confirms prior findings showing accurate predictions by the ACS-SRC in Acute Care, Colorectal, and General Surgery.

Cardiothoracic surgery also had high predictive accuracy in the current study, however previous studies suggested the opposite. Hers et al. evaluated the ACS-SRC's predictive ability in 234 patients undergoing endovascular or open aortic aneurysm repair [22]. The calculator was only predictive when assessing the risk of readmission and sepsis. All other complications reported by the calculator were poorly predicted [22]. The authors concluded that the calculator has a poor predictive ability when used in the setting of complex procedures [23,24]. Taking these findings in conjunction with our work, it suggests that with a larger sample, perhaps the ACS-SRC is suitable in cardiothoracic surgery.

This study found that the ACS-SRC does not accurately predict complications in Spine, Orthopaedics, Gynecology, ENT, Urology, and Surgical Oncology, which is congruent with prior work. Within Spine surgery, a study by Narain et al. discussed the predictive ability of the ACS-SRC in the context of anterior lumbar interbody fusions (ALIF). The authors found poor predictive ability of the ACS-SRC and attributed it to the lack of inclusion of important ALIF risk factors such as nutritional status, anemia, and weight loss [25,26].

Although, our study showed the ACS-SRC to underpredict complications in Orthopaedic Surgery; there are conflicting results in the literature. Edelstein et al. assessed the ACS-SRC predictive accuracy in 1764 Medicare patients undergoing total hip arthroplasty (THA) or total knee arthroplasty (TKA). They found the ACS-SRC accurately predicted any complication, cardiac complications, pneumonia, and adverse discharge rates [27]. Wang et al. reported an accurate prediction of reoperation rate in a cohort of 410 elderly hip fracture patients. For both these studies, however, death and serious complication rates were not well-predicted by the ACS-SRC [27,28]. While past work focused solely on arthroplasty patients, our sample combines this with orthopaedic oncology and a joint infection population, providing a broader view of the field. This difference within study populations among orthopaedic surgery may explain the variability of the ACS-SRC.

In Gynecology, multiple studies have reported on the poor-predictive ability of the ACS-SRC. Hamade et al. examined the utility of the calculator in hysterectomy patients and concluded poor predictive ability for all outcomes due to calculator over-prediction [29]. Rivard et al. evaluated the ACS-SRC across numerous gynecologic oncology procedures and noted that these consisted of only 5.3 % of cases included in the NSQIP database. The authors ascribed the poor predictive ability of ACS-SRC to case complexity and the inability to enter multiple CPT codes into the calculator. This likely reduces patient or procedure-specific nuances [19]. Both gynecologic studies commented on the poor pre-operative nutritional status of their patient population and emphasized the addition of this factor to the calculator pre-operative metrics [19,29].

Within ENT, Vosler et al. compared free flap (FF) reconstruction and non-free flap (NFF) reconstruction procedures [30]. They found the ACS-SRC accurately predicted NFF complications but not FF. For the NFF group, all metrics listed by the ACS-SRC were accurately predicted, whereas the FF group only had 8/12 outcomes were accurately predicted. They postulated this was due to the technical challenge of the FF procedure, increased time under anesthesia, and extended post-operative stay. [30]. Similar to Rivard et al. in Gynecology, they concluded inherent procedural complexity limits the ACS-SRC's predictive ability in ENT [19,30]. While there is no significant difference between predicted and observed values in the current study, the calculator still underpredicts by 4.5 % which may be relevant clinically.

Urology was significantly underpredicted in our study and similarly shows mixed results in the literature. Of note, much of the prior literature applies the ACS-SRC in oncological procedures with gross underprediction of many complications [15,31,32]. One study by Blair et al. evaluated the calculator in a partial nephrectomy cohort and reported significant underestimation for any complications, SSI, UTI, and LOS. In contrast, they found it over-estimated serious complications [15]. Similar to ENT and Spine Surgery, Blair et al. faults procedure complexity and patient pre-operative nutrition status for the discrepancy in urological procedures [15,32]. With the addition of pre-operative variables such as nutritional status or multiple CPT codes, the calculator may improve prediction deficits [15,19,29].

This work is not without limitations. Studies that only reported statistical metrics instead of proportions were excluded due to limited data availability. These included studies that only reported brier score, c-statistic, or AUC. This resulted in omitted data that may have affected our conclusions, which is a known limitation of metanalyzes. Finally, this study is reliant on accurate data reporting, and is therefore subject to each article's listed limitations.

While the ACS-SRC can be used for certain surgical fields, the aforementioned studies reveal substantial limitations if it is applied indiscriminately across all surgical specialties. While the calculator encompasses many institutions, each can choose, by specialty, which data to submit. This may lead to robust data from well-predicted fields (General Surgery, Acute Care Surgery), but limited data from poorly predicted fields (Spine, ENT, Gynecology, and Urology). Additionally, procedure complexity may hinder accurate prediction, which has been discussed by many specialties as a limitation in using this calculator. Multiple studies have identified the need for inputting multiple CPT codes, in the hopes that it will account for greater specificity and complexity [19]. Furthermore, the ACS-SRC lacks important pre-operative metrics such as nutritional status [25,26]. Inclusion of these features may improve post-operative complication rates. Specialty-specific extensions of the ACS-SRC would similarly provide valuable insight.

The ACS-SRC may be a valuable tool in pre-operative planning and clinical decision-making for patients but with limited generalizability. We caution surgeons applying the calculator in the subspecialties mentioned here, where accuracy is limited. On these occasions, a multifactorial approach, including physician experience and unique patient characteristics, should provide context to inform decision-making. With many changing reimbursement structures, knowledge of patient-specific complication risks can provide value to patients, surgeons, institutions, and payers [33].

## CRediT authorship contribution statement

**Alyssa M. Goodwin:** Data curation, Formal analysis, Funding acquisition, Investigation, Methodology, Project administration, Resources, Validation, Visualization, Writing – original draft, Writing – review & editing. **Steven S. Kurapaty:** Formal analysis, Investigation, Methodology, Software, Supervision, Writing – original draft, Writing – review & editing. **Jacqueline E. Inglis:** Data curation, Methodology, Writing – review & editing. **Srikanth N. Divi:** Writing – review & editing. **Alpesh A. Patel:** Writing – review & editing. **Wellington K. Hsu:** Conceptualization, Supervision, Validation, Writing – review & editing.

## Declaration of competing interest

The authors declare that they have no known competing financial interests or personal relationships that could have appeared to influence the work reported in this paper.
